# Impaired Growth and Force Production in Skeletal Muscles of Young Partially Pancreatectomized Rats: A Model of Adolescent Type 1 Diabetic Myopathy?

**DOI:** 10.1371/journal.pone.0014032

**Published:** 2010-11-17

**Authors:** Carly S. Gordon, Antonio S. Serino, Matthew P. Krause, Jonathan E. Campbell, Enzo Cafarelli, Olasunkanmi A. J. Adegoke, Thomas J. Hawke, Michael C. Riddell

**Affiliations:** 1 School of Kinesiology and Health Science, York University, Toronto, Ontario, Canada; 2 Muscle Health Research Centre, York University, Toronto, Ontario, Canada; 3 Department of Pathology and Molecular Medicine, McMaster University, Hamilton, Ontario, Canada; Universidad Europea de Madrid, Spain

## Abstract

This present study investigated the temporal effects of type 1 diabetes mellitus (T1DM) on adolescent skeletal muscle growth, morphology and contractile properties using a 90% partial pancreatecomy (Px) model of the disease. Four week-old male Sprague-Dawley rats were randomly assigned to Px (n = 25) or Sham (n = 24) surgery groups and euthanized at 4 or 8 weeks following an *in situ* assessment of muscle force production. Compared to Shams, Px were hyperglycemic (>15 mM) and displayed attenuated body mass gains by days 2 and 4, respectively (both P<0.05). Absolute maximal force production of the gastrocnemius plantaris soleus complex (GPS) was 30% and 50% lower in Px vs. Shams at 4 and 8 weeks, respectively (P<0.01). GP mass was 35% lower in Px vs Shams at 4 weeks (1.24±0.06 g vs. 1.93±0.03 g, P<0.05) and 45% lower at 8 weeks (1.57±0.12 vs. 2.80±0.06, P<0.05). GP fiber area was 15–20% lower in Px vs. Shams at 4 weeks in all fiber types. At 8 weeks, GP type I and II fiber areas were ∼25% and 40% less, respectively, in Px vs. Shams (group by fiber type interactions, P<0.05). Phosphorylation states of 4E-BP1 and S6K1 following leucine gavage increased 2.0- and 3.5-fold, respectively, in Shams but not in Px. Px rats also had impaired rates of muscle protein synthesis in the basal state and in response to gavage. Taken together, these data indicate that exposure of growing skeletal muscle to uncontrolled T1DM significantly impairs muscle growth and function largely as a result of impaired protein synthesis in type II fibers.

## Introduction

Type 1 diabetes mellitus (T1DM) is characterized by complete or near-complete insulin deficiency resulting from an autoimmune-mediated selective destruction of the pancreatic β-cells. Adults with long-standing and poorly controlled T1DM often present with a number of disease-related complications including neuropathy, nephropathy, retinopathy and cardiovascular disease [1]. While it is possible to have these micro- and macrovascular complications in youth with T1DM, the incidence is rare [2]. Given that skeletal muscle is the largest organ for glucose disposal, ensuring the maximal growth and development of muscle may improve the capacity for blood glucose disposal and thereby attenuate other diabetic complications, a critical strategy for those with T1DM. At the very least, maximizing muscle mass would aid in the functional performance and fitness of those living with the disease.

For many individuals, T1DM onset occurs in childhood and there is often a protracted period of time before diagnosis. Even following the diagnosis of T1DM, glycemic management is difficult and very often suboptimal in childhood and adolescence [3]. Unfortunately, T1DM onset in youth coincides with a rapid growth phase of skeletal muscle and atrophic stimuli placed on the muscle during this time can lead to a rapid and irreversible remodeling process, resulting in lifetime of reduced muscle mass and physical capacity [4], [5]. Currently, our understanding of the effects of T1DM on the skeletal muscle of pediatric populations is vague though some evidence exists that the skeletal muscle of young adults with T1DM is compromised (e.g. relative muscle fiber atrophy, sarcomere destruction) even before evidence of peripheral neuropathy is observed [6]–[9].

The purpose of this study was to define the temporal effects of T1DM on adolescent rodent skeletal muscle growth, morphology and contractile characteristics in a non-genetic, non-pharmacological model of disease, the 90% partial pancreatectomy (Px) rat. For this, we examined two separate time points of diabetes duration to identify the early and late alterations that occur in response to the hypoinsulinemic/hyperglycemic state. We hypothesized that Px rats would display impaired skeletal muscle growth and functional capacity and that these impairments would become more significant with the duration of hypoinsulinemia/hyperglycemia. Furthermore, we hypothesized that lowered rates of protein synthesis would largely be responsible for the attenuated muscle growth within growing T1DM rats. The results of this study support our hypothesis that exposure of young muscle to a T1DM environment results in impairments in skeletal muscle growth, a finding that intensifies with increasing disease duration and is particularly profound in type II muscle fibers. Our findings also support the hypothesis that this attenuated growth is mediated in part by impaired protein synthesis, as activation of the mammalian target of rapamycin (mTOR) pathway is markedly reduced as early as 4 weeks after the development of T1DM. These findings help define the temporal alterations occurring to growing skeletal muscle in response to T1DM and aid in defining the underlying mechanisms of impaired muscle mass growth and contractile function.

## Methods

### Ethics Statement

All experiments were approved by the York University Animal Care Committee in accordance with Canadian Council for Animal Care guidelines (protocol #2007-22).

### Animal Characteristics

Young, male Sprague Dawley rats (∼age 1 month, 45–55 g) were purchased from Charles River Laboratories (Montreal, QC, Canada) and allowed to acclimate for 6 days upon arrival. The animals (100–120 g) were then randomly assigned to one of four groups: 4 week Sham pancreatectomy (n = 10), 4 week Px (n = 14), 8 week Sham (n = 14), and 8 week Px (n = 11). A group of 8 healthy animals four week old male Sprague Dawley rats (mean weight  = 104±0.9 g; fed blood glucose 6.08±0.23 mM) were also sacrificed following an overnight fast to serve as a baseline for growth. The two different time periods of hyperglycemia (i.e. 4- and 8-weeks) were used to determine the time course of myopathic changes in this T1DM model. Each time point refers to the time from surgery to the day of harvest. All animals were housed in pairs and provided with standard rat chow and water *ad libitum*. The animal room was maintained between 22–23°C, 50–60% humidity and a 12 h/12 h light-dark cycle.

On the seventh day following arrival (day 0), animals underwent surgery to either remove 90% of the pancreas (Px) or to have a Sham procedure, as described previously [10]. For this, rats were anesthetized with 2% inhaled isofluorane and an incision was made extending from the xyphoid process of the sternum to hip level. Using cotton tip applicators, the splenic, gastric and duodenal regions of the pancreas were removed leaving all major vessels intact to not compromise the surrounding organs. The area within 2 mm of the common bile duct, extending from the duct to the first part of the duodenum, was left intact and classified as the residual pancreas (∼10% of the initial pancreatic mass). Sham animals underwent the same surgical procedure but with no pancreas removal, only titillation of the pancreas with a cotton tip applicator. All rats recovered in clean, standard rodent cages in groups of two. Blood glucose levels were measured via tail nick, 4 times (at 09:00 h) in the first week to monitor critical changes in blood glucose following surgery. Thereafter, measurements were taken 2 times a week to follow glycemic levels but minimize animal handling and stress. On day 27, fasting glycemia (after an 18 hour fast) was also measured. Body mass was measured three times per week (09:00 h) with animals in the fed state for the duration of the protocol. On day 25, fed plasma samples were obtained at 08:00 h for basal corticosterone (CORT; ImmunoChem double antibody CORT RIA, ICN Biomedicals Inc., Costa Mesa, CA) and insulin (90080 Ultra Sensitive Rat Insulin ELISA Kit, Crystal Chem Inc., IL) concentrations in the 4 week group only. During the 3^rd^ and 6^th^ week, food and water consumption were assessed over a 24 hour period, based on 2 animals per cage.

### In Situ Muscle Stimulation

Maximum peak force (Fmax) and fatigability of the gastrocnemius plantaris soleus muscle complex (GPS) was determined at 4 and 8 weeks after surgery. Following an overnight fast, animals were sedated with a ketamine/xylazine cocktail, via intraperitoneal (IP) injection prior to surgery. The surgical procedure and muscle stimulation via the sciatic nerve was performed following adaptation to the original protocol described previously by Burke et al., 1973 [11]. Briefly, the surgical procedure consisted of carefully removing the skin around the ankle and leg, followed by removal of the hamstrings to expose the GPS. The sciatic nerve was carefully dissected and isolated with surgical thread and cut 1.5 cm from its disappearance into the GPS to prevent retrograde stimulation. A metal pin was sutured to the Achilles tendon for attachment to the force transducer, and the trochanters of the femur were pinned to prevent movement of the leg during isometric contractions. Blood flow to the muscle was left undisturbed. The muscles were bathed with saline and wrapped in plastic wrap to prevent desiccation. Optimal voltage and muscle length was determined for each animal by generating single twitch contractions at increasing voltages and muscle lengths until no increase in single-twitch force production were observed. The muscle length (L_0_) and voltage that generated the highest single twitch amplitude was then used throughout the entire stimulation protocol. The pulse duration was set to 1 msec for all twitch and tetanic contractions. The stimulation protocol consisted of a force frequency curve to determine F_max_, followed by a 2 min stimulation period to determine fatigue resistance. F_max_ was determined using brief, repeated stimulations at increasing pulse frequencies until no further increase in force was observed (evident by either a plateau or drop off in force). The greatest force achieved for each animal using this protocol was considered the F_max_. Following a two minute recovery period after F_max_ determination, muscle fatigue rate was determined over a 2 minute period of intermittent contractions, stimulating the muscle for 3 seconds on and 3 seconds off. Stimulation was given via the sciatic nerve at a frequency that generated 50% F_max_. Twitch amplitude, time to peak tension and half-relaxation time were also determined before the fatigue protocol. Time to peak tension was defined as the time elapsed from the base to the peak of a single twitch. Half-relaxation time was defined as the time elapsed from the peak of a single twitch to the point of the twitch amplitude returning halfway to baseline. All muscle function data were collected through an AD Instruments Bridge Amp and Powerlab 4/30, and analyzed with Chart5 PowerLab software (ADInstruments, Inc., Colorado Springs, CO).

### Tissue Collection, Blood and Enzyme Activity Analyses

Following the stimulation protocol, animals were euthanized via decapitation with blood and tissues harvested immediately. Muscles were extracted, the soleus was removed from the GPS and tissues were weighed using a standard laboratory bench scale. The length of the GP was taken using a micrometer. Portions of muscle tissues from the unstimulated leg were mounted on a 2 cm diameter cork base using mounting medium and then quick-frozen in liquid nitrogen-cooled isopentane. Decapitation blood was collected and plasma was separated and analyzed for fasted non-esterified free fatty acids (NEFA; HR Series NEFA-HR2 kit; Wako Diagnostics, Richmond, VA) and insulin (90080 Ultra Sensitive Rat Insulin ELISA Kit, Crystal Chem Inc., IL) concentrations. Cytochrome c oxidase (COX) activity of the GP and soleus muscles were measured from liquid nitrogen snap frozen samples on the contralateral limb based on previously reported procedures [12].

### Histochemistry

To identify skeletal muscle fiber type, a metachromatic myosin ATPase stain was performed on 10 µm thick cross sections of the GP muscles using a modified Ogilvie and Feeback protocol [13]. Sections were pre-incubated in an acidic buffer (pH = 4.40) to differentially inhibit myosin ATPases within the different fiber types. In this protocol using light microscopy, type I fibers appear dark blue, type IIa appear pink and type IIb and IId are not discernible from each other and classified as IIb/d. These fibers appear bluish-purple. The mixed gastrocnemius and plantaris regions of both the 4 and 8 week group were identified on each section and a representative image of each muscle region was acquired for analysis. Over 200 fibers were counted per microscope image, per animal, to determine fiber type composition in a given area. Average fiber area was quantified based on the average area of ∼20 fibers for each fiber type per image [14]. Quantification analysis was performed with Adobe Photoshop CS version 8.0 and reported in µm^2^. All images were acquired with a Nikon Eclipse 90i microscope and Q-Imaging MicroPublisher 3.3 RTV camera with Q-Capture software.

Succinate dehydrogenase (SDH) activity was assessed using a histochemical analysis and expressed in relative optical density to Shams, as previously described [14]. The same muscle regions of the mixed gastrocnemius that were used for fiber typing were used for SDH activity determination. For this, serial sections were used to directly compare levels of SDH in each fiber, to each fiber type. SDH activity is reported as average optical intensity of SDH in each fiber.

### Markers of Mammalian Target of Rapamycin (mTOR) Signaling, Fractional Protein Synthesis Rates and Protein Degradation

Eukaryotic initiation factor 4E binding protein 1 (4EBP1) and p70/p85-S6 protein kinase (S6K1) are key components in the mRNA translation machinery for protein synthesis. Both 4EBP-1 and S6K1 are downstream phosphorylation targets of the mammalian target of rapamycin (mTOR) that stimulate mRNA translation initiation and thus muscle protein synthesis in the post-absorptive state. To determine if mTOR signaling was impaired in Px animals in response to feeding, another group of age matched Px (n = 8) and Sham (n = 6) rats were administered a leucine gavage 4 weeks post surgery in the fasted state (18 hours) to mimic an acute feeding stimulus [15]. For the protein feeding stimulus, a solution of 20 g of leucine (L-Leucine, cat # L8912, Sigma-Aldrich Canada, Oakville, ON, Canada) was dissolved in 1L distilled water. Half of the rats in each group (n = 4 and 3 in Px and Shams, respectively) were administered, via oral gavage, standardized volumes of the solution (0.48 g of L-Leucine per kg of body weight) while the other half were administered an equivalent volume of water (24 mL/kg). This amount of leucine intake is representative of what is thought to stimulate protein synthesis in young rats [15]. Thirty minutes following oral gavage, the animals were killed by decapitation and the gastrocnemius was quickly removed, separated from the plantaris and flash frozen in liquid nitrogen for future analysis.

For analysis of 4EBP-1 and S6K1, mixed gastrocnemius muscle (100 mg) was homogenized on ice in 10 parts buffer (in mM: 20 HEPES, 2 EGTA, 50 NaF, 100 KCl, 0.2 EDTA, 50 glycerolphosphate, pH 7.4) supplemented with 1 mM DTT, 1 mM benzamidine, 0.5 mM sodium vanadate, protease inhibitor cocktail and phosphoatase inhibitor 2 cocktail (P8340, P5726; Sigma-Aldrich Canada, Oakville, ON, Canada) and then centrifuged 30 minutes at 13,000 RPM and at 4°C [16]. Samples were quantified for protein concentration using the BioRad Protein Assay Kit (BioRad, Canada). Acrylamide gels were prepared by using the Bio-Rad electrophoresis equipment, 15% for 4EBP-1 and 10% for S6K1 and ubiquitinated proteins. Equal amounts of protein were loaded into each well. The gels were run at 120 V for 2 hrs and then transferred onto PVDF membranes at 85 V for 3 hr. Nonspecific sites were blocked by incubation in 5% BSA in TBS-T and then incubated overnight at 4°C with primary antibody. For analysis of 4EBP-1, primary antibody (#9644; Cell Signaling Technologies, New England Biolabs, Pickering, ON, Canada) was applied in 1∶10,000 ratio. For analysis of phosphorylated S6K1 (Thr 389) and total S6K1, primary antibody (#9234, #9202; Cell Signaling Technologies, New England Biolabs, Pickering, ON, Canada) was applied in a 1∶1,000 ratio.

Protein synthesis was measured in mixed gastrocnemius muscle samples in 4 week animals using the flooding dose method developed by Garlick et al. [17]. For this, L-[2,3,4,5,6-3H] phenylalanine (TRK648, GE Healthcare Canada) was combined with unlabelled phenylalanine (150 mM in PBS, #5202 EMD Chemicals Canada) to give a 50 µCi/ml solution of L-[2,3,4,5,6-3H] phenylalanine to be injected into the animals. The muscle powder of ∼100 mg of tissue was homogenized in 1 mL of cold 2% HClO_4_ and centrifuged at 2000 g for fifteen minutes. The supernatant was then collected and ∼0.5 mL of saturated potassium citrate was added. This sample was centrifuged at 2000 g for fifteen minutes and was used to determine the specific radioactivity of free phenylalanine in the precursor pool. Protein-bound phenylalanine was obtained by washing the pellet with 4 mL of HClO_4_ four times and then hydrolyzing the protein in 5 mL of 6 M- HCl for 24 hours at 110°C. HCl was removed via evaporation using a vacuum system and the amino acids were re-suspended in 1 mL of 0.5 M sodium citrate. An enzymatic conversion to β-phenethylamine was then performed to account for [H3] phenylalanine that may have been converted into [H3] tyrosine. For this, ∼1.25 mL of the supernatant and 0.8 mL of hyrosolate was incubated with 0.20 mL or 0.285 mL, respectively, from a 2units/mL L-tyrosine decarboxylase solution (cat # T7927, Sigma-Aldrich Canada) for 20 hours at 50°C. β-phenethylamine was extracted by adding 0.5 mL of 3 M NaOH and 5 mL of chloroform: n-heptane (1∶3). Samples were shaken and centrifuged at 500 g for 5 minutes and the organic layer from these samples was removed and 2.5 mL of chloroform and 1 mL of 0.1 M H2SO4 was added. Again samples were shaken and centrifuged at 500 g for 5 minutes and then the upper aqueous phase was removed. Subsequently, both the supernatant and the pellet were assayed for phenethylamine using a modified version of a method developed by Suzuki and Yagi [18]. For this, 0.1 mL of 2 mM L-leucyl-L-alanine, 0.5 mL of 1 M potassium phosphate and 0.2 mL of 50 mM ninhydrin were added to specific volumes of the supernatant and the pellet. Samples were incubated for 30 minutes at 60°C and then cooled in ice for fifteen minutes. Fluorescence was measured using a 96 well plate reader (Synergy HT Multi-Mode Microplate Reader, BioTek, USA). Finally, specific activity was calculated by dividing the radioactivity of each sample by its concentration of phenethylamine [(DPM/mL)/(nmol/mL)]. Fractional rates of protein synthesis (ks) were determined using the formula ks =  (SB * 100)/(SA * t), where t is the time interval between the time of injection and the freezing of sample in liquid nitrogen expressed in days, SB is the specific activity in the protein bound amino acids and SA is the specific activity in the precursor pool.

For degradation analysis, we assessed the ubiquitin proteolytic system as it is the main intracellular proteolytic pathway in muscle. Because proteins to be degraded are first ubiquitinated prior to being degraded by the 26S proteasome, increase in the amount of ubiquitinated protein reflects increase in the activity of the pathway and correlates with increased muscle proteolysis [19]. For this analysis, primary antibody (sc-8017; Santa Cruz Biotechnology, Santa Cruz, CA) was applied in 1∶1,000 ratio. Secondary antibody (ab6789, ab6721; Abcam Inc., Cambridge, Massachusetts, U.S.A) was applied in 1∶10,000 ratio. Enhanced chemiluminescent detection (WBKL S05 00; Millipore, Etobicoke, Ontario, Canada) and Kodak Image Station were used to visualize the bands. Results are expressed as a fraction of phosphorylated to total protein. Results for 4EBP-1 are expressed as the fraction of γ (the most phosphorylated) to the total of α, β and γ. Phophorylated S6K1 was expressed relative to total S6K1.

### Data analysis

A two-way mixed analysis of variance (ANOVA), followed by a Fisher post hoc test if necessary, was used for analysis of blood glucose, body mass, food consumptions and water intake over the experimental period. Since we had data for both 4- and 8- week animals for the above parameters over the first four weeks, these data were pooled for analysis and graphical representation. ANOVAs were also conducted on force-frequency curves, fatigue curves, muscle twitch tension, rise time and half relaxation time and M wave amplitude. Two-way factorial ANOVAs were used for the analysis of organ/muscle masses, absolute and relative F_max_, fiber area and muscle SDH activity. Chi squared tests are performed on muscle mass and fiber area growth between 4 and 8 weeks. For within the same time periods (i.e. 4 or 8 weeks), t-tests were used for comparisons between Px and Sham rats for the following variables: GP muscle length, cytochrome c oxidase activity; and hormone concentrations. Scion Image Software (Scion, Frederick, MD) was used to measure the optical density of S6K1 and 4EBP-1 protein expression and a one-way ANOVA was used for analysis. Statistical analysis was completed using Statistica 6.0 statistical software (StatSoft®, Tulsa, OK), with P≤0.05 as the criterion for statistical significance. All data are expressed as mean ± standard error of mean (SEM).

## Results

### Animal Characteristics

**Figure 1:**
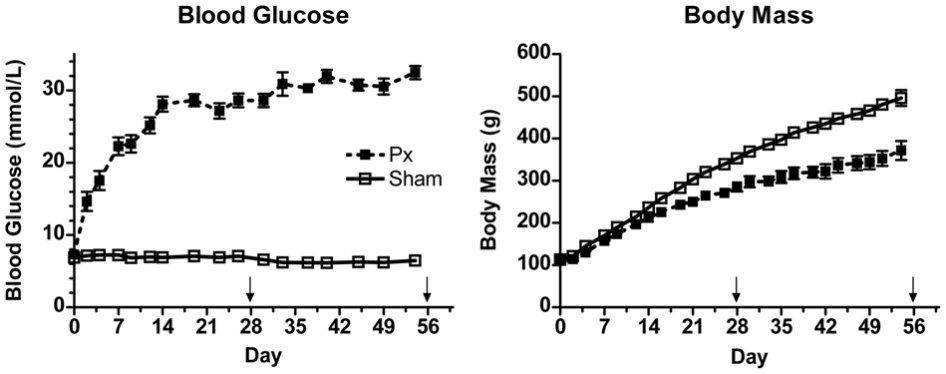
Fed blood glucose levels and body masses in Px and Sham rats following surgery. Blood glucose level was higher in Px vs. Shams on day 2 following surgery and throughout the protocol (P<0.05). Body mass was lower in Px vs. Shams at day 4 following surgery and throughout the rest of the protocol (P<0.05). Arrows indicate time of harvest for 4 week (n = 14 Px; 9 Shams) and 8 week (n = 11; 15 Shams) groups.

Whole blood glucose concentrations and body masses in the fed state in Px and Sham groups over the 8 weeks of study are shown in Figure 1. Px rats had elevated blood glucose levels two days after surgery (P<0.05) and values remained elevated (>25 mM) throughout the experimental period. On day 28, at the time of harvest of the 4 week groups, ∼18 hour overnight fasted whole blood glucose concentration was also higher in Px vs. Shams (measured in gavaged animals only: 8.1±1.3 mM, n = 8 vs. 4.3±0.2 mM, n = 6, P<0.001). Body mass of Px rats was significantly lower than Shams by day 4 of the protocol and remained significantly less than Shams throughout the rest of the experimental period (Figure 1). In the fasted state, Px rats weighed 26% less than Sham-surgery rats at 4 weeks (Sham: 340±6 g vs. Px: 251±9, P<0.05) and 33% less by 8 weeks after T1DM development (Sham: 466±8 g vs. Px: 311±16 g, P<0.05).

Food and water intake at 3 and 6 weeks post surgery illustrate that Px rats were hyperphagic (3 weeks: Sham: 62±2 vs. Px  = 97±12 g/day, P<0.05; 6 weeks: Sham: 57±2 vs. Px: 105±4 g/day, P<0.05) and polydipsic (3 weeks: Sham: 82±3 vs. Px: 416±29 ml/day, P<0.05; 6 weeks: Sham: 68±7 vs. Px: 452±32 ml/day, P<0.05) compared to Sham surgery rats.

GP, tibialis anterior and soleus masses at 0-, 4- and 8- weeks in Px and Shams are shown in Table 1. At both 4 and 8 weeks of T1DM, all muscles measured in Px rats weighed significantly less than Shams (all P<0.05). Although both Px and Sham animals had greater muscle masses at 8 weeks when compared to 4 weeks (both P<0.05), indicating that growth had occurred, the percent change in GP mass was significantly greater in the Shams than in the Px animals (Chi square, P<0.05). The percent change in mass of the soleus and tibialis anterior muscles were not significantly different between Shams and Px. GP length was similar between groups at 4 weeks (Sham: 1.26±0.08 vs. Px: 1.23±0.07 cm; P>0.05), however, Px muscle length was significantly less than Shams at 8 weeks (Sham: 3.26±0.06 vs. Px: 3.00±0.09 cm; P<0.05). Compared with Shams, Px rats also had severe reductions in retroperitoneal fat mass at 4 weeks (2.79±0.31 g vs. 0.22±0.09 g) and at 8 weeks (5.27±0.45 g vs. 0.19±0.16 g) (both P<0.001). Similarly, epididymal fat pad mass was less in Px than in Shams at 4 weeks (0.68±0.08 g vs. 2.69±0.33 g) and at 8 weeks (1.06±0.14 g vs. 5.21±0.29 g) (both P<0.05).

**Table 1:** Anthropometric data for Px and Sham groups at 4 and 8 weeks.

Tissue	0 Weeks	4 Weeks		8 Weeks		Sham change wks 4–8	Px change wks 4–8
	Sham mass (g)	Sham mass (g)	Px mass (g)	Sham mass (g)	Px mass (g)		
Gastrocnemius-plantaris	*0.51±0.01*	*1.93±0.03 ^#^*	*1.24±0.06 ^#,^* *	*2.80±0.06* φ	*1.57±0.12* *φ	146%	127% δ
Tibialis anterior	*0.17±0.01*	*0.61±0.01 ^#^*	*0.40±0.02 ^#,^* *	*0.82±0.02* φ	*0.48±0.03* *φ	139%	121%
Soleus	*0.04±0.001*	*0.148±0.011 ^#^*	*0.098±0.004 ^#,^* *	*0.196±0.006* φ	*0.124±0.008* *φ	135%	127%
Epididymal fat	*0.31±0.03*	*2.69±0.33 ^#^*	*0.68±0.08 ^#,^* *	*5.21±0.29* φ	*1.06±0.14* *	165%	157% δ
Retroperitoneal fat	*0.26±0.02*	*2.79±0.31 ^#^*	*0.22±0.09 ^#,^* *	*5.27±0.45* φ	*0.19±0.16* *	189%	89% δ

Note:

# indicates 4 week significantly greater than baseline at P<0.05.

*indicates that Px is significantly less than Sham for the same time point at P<0.05.

φ indicates that 8 week values are significantly higher than at 4 weeks within the same group at p<0.05.

δ indicates a significant change in growth between 4 and 8 weeks.

At 4 weeks post-surgery, fasted insulin concentrations were similar between Px and Shams, while at 8 weeks post-surgery the Px rats had ∼50% less fasted insulin levels than Sham rats (Table 2). In Px rats, insulin levels failed to increase in response to feeding, while in Shams, levels increased more than three fold in response to feeding (Table 2). In the fed state, basal (0800 h) glucocorticoid concentrations at ∼3.5 weeks post-surgery tended to be elevated in Px compared to Shams, though not significantly so (P = 0.07; Table 2), likely because of the high variance in the Px group. In the fasted state, Px rats had less circulating NEFA compared to Sham surgery rats at both 4 and 8 weeks (P<0.05; Table 2).

**Table 2:** Fed and fasted plasma hormone concentrations at 4 and 8 weeks.

	Week	Sham	Px
Basal fasted insulin concentration (ng/ml)	4	*0.54±0.08*	*0.47±0.08*
	8	*0.80±0.10*	*0.42±0.04* *
Basal fed insulin concentration (ng/ml)	4	*5.79±0.59*	*0.80±0.20* *
Basal fed glucocorticoid concentration (ng/ml)	4	*19.3±3.03*	*69.3±22.8*
Fasted non-esterified fatty acids (mM)	4	*0.44±0.07*	*0.22±0.04* *
	8	*0.39±0.03*	*0.28±0.25* *

T-tests were performed on plasma hormone concentrations.

*indicates that Px is significantly less than Sham for the same time point at p<0.05. Basal glucocorticoid levels tended to be higher in Px than Shams (P = 0.07).

### Muscle Function

**Figure 2:**
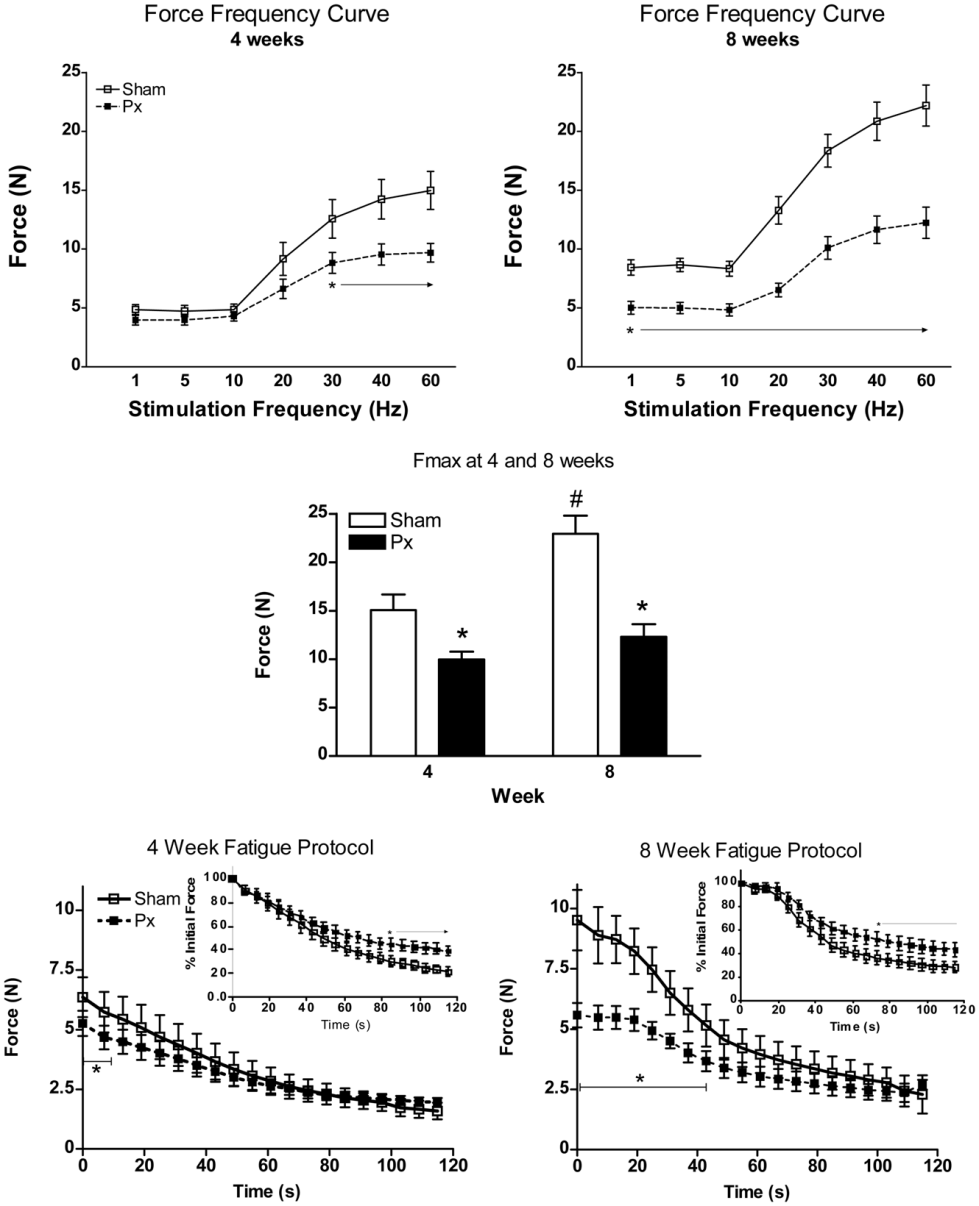
In situ muscle stimulation force frequency curves (A), Fmax (B) and fatigue index curves (C) at 4 and 8 weeks in Px and Sham rats. At 4 weeks, Px rats displayed lower contractile force compared to shams at frequencies ≥30 Hz (upper left panel). At 8 weeks, Px showed less force generation at all stimulation frequencies (upper right panel). Maximal tetanic force (Fmax) was lower in Px compared to Sham rats at both 4 and 8 weeks (center panel) and only increased with time in the Shams (#, P<0.05). Absolute contractile force during the 2 minute fatigue protocol was less at the start of the test in Px compared to Shams at both 4 and 8 weeks (bottom left and right panels), but values reached similar fatigue endpoints by the end of 2 minutes. When expressed as a percentage of initial force (insets), Px had a smaller decline in force over time at both 4 and 8 weeks. * indicates Px different from Sham at p<0.05; # indicates greater than 4 weeks (P<0.05).

Figure 2 shows the force frequency curves and fatigue profiles at 4 and 8 weeks in the Px and Sham rats. At 4 weeks, Px rats had attenuated absolute tetanic contractile force production, particularly at higher stimulation frequencies (i.e. ≥30 Hz), compared to Shams (Figure 2, upper left panel). The dynamics of force production, when expressed relative to Fmax in each group, were similar between Sham and Px rats (Figure S1). As disease duration progressed, the Px rats had a reduced tetanic contractile force production at all stimulation frequencies tested compared to Shams (Figure 2, upper right panel), although the dynamics of force production remained similar between groups (Figure S1). In the Shams, there were increases in absolute force generation at all stimulation frequencies between 4 and 8 weeks (P<0.05), indicative of increased strength with maturation, while no gain in force generation was observed between 4 and 8 weeks in Px rats (Figure 2).

Absolute maximal tetanic force production (F_max_) at 4 and 8 weeks is shown in Figure 2, middle panel. F_max_ in Px rats was ∼30% lower than Shams at 4 weeks and ∼50% lower at 8 weeks. Moreover, F_max_ increased by ∼50% in Shams between 4 and 8 weeks but did not change significantly with time in Px rats (4 week: Sham: 15.0±1.62 N vs. Px: 9.97±0.81 N; 8 week: Sham: 22.2±1.74 N vs. Px: 12.3±1.31 N, group by time interaction, P<0.05). When expressed relative to the muscle mass of the GPS (N/g muscle mass), there were no differences in F_max_ between groups nor between time points (4 week Sham  = 7.86±0.91; 4 week Px  = 7.95±0.91; 8 week Sham  = 6.83±0.51; 8 week Px  = 6.94±0.48 N/g).

Absolute force generation during the two minute fatigue protocol is shown in Figure 2, lower panels. As expected, the absolute force production was initially lower in Px than in Shams at both 4 and 8 weeks, given that the fatigue protocol was designed to elicit initial contractions of 50% F_max_. At the end of the two minute fatigue protocol, the level of fatigue observed between groups was similar. When expressed relative to initial force (see Figure 2, lower panel insets), Px rats displayed an attenuated force decline (4 weeks: 61±5% reduction in force; 8 weeks: 47±3% reduction in force) compared to that observed in Shams at both 4 and 8 weeks (4 weeks: 78±4% reduction in force; 8 weeks: 72±6% reduction in force; p<0.05 both time points).

Muscle twitch rise time is commonly used as a marker of intracellular calcium release. Muscle compound action potential (i.e. M wave) is an index of neuromuscular propagation, or the process involved in converting an axonal action potential into a muscle fiber action potential. Muscle twitch characteristics and M wave amplitude in Px and Shams at 4 and 8 weeks are shown in Table 3. Absolute twitch tension, time to peak tension and half-relaxation time were all similar between Px and Shams at 4 weeks. At 8 weeks, time to peak tension and half-relaxation time were less in Px than in Shams (both P<0.05).

**Table 3:** Muscle twitch characteristics at 4 and 8 weeks.

	Week	Sham	Px
Absolute twitch tension (N)	4	*6.12±0.62*	*5.64±0.93*
	8	*6.68±0.99*	*5.23±0.98*
Time to peak tension (ms)	4	*30.3±3.55*	*31.3±2.49*
	8	*36.8±2.78*	*29.5±0.97* *
Half- relaxation time (ms)	4	*32.1±6.93*	*35.2±5.84*
	8	*40.3±7.69*	*24.0±1.74* *

Note:

*indicates that Px is significantly less than Sham for the same time point at p<0.05.

### Muscle Fiber Cross-Sectional Area

**Figure 3:**
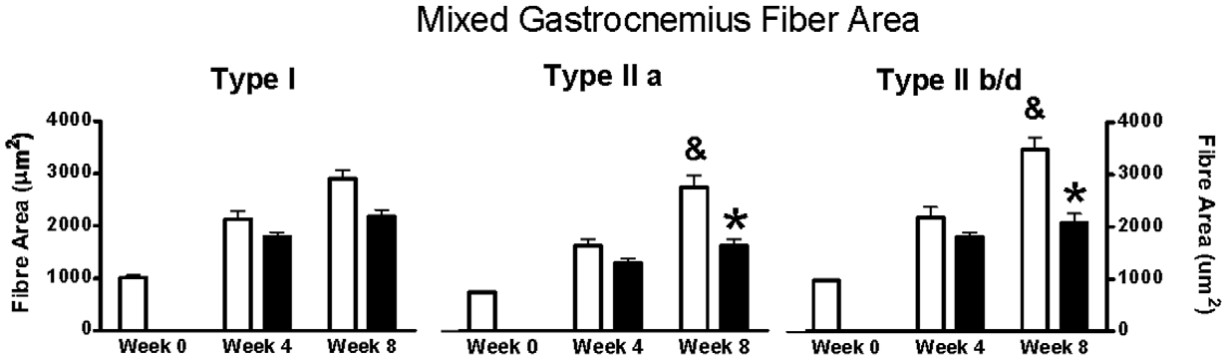
Muscle fiber areas in mixed gastrocnemius at baseline (time 0) and at 4 and 8 weeks in Px and Shams. In all fiber types, there were main effects for group, main effects for time, and group by time interactions (all P<0.05). & indicates significantly greater than week 4 (P<0.05). * indicates Px lower than Shams (P<0.05).

The cross sectional areas of type I, IIa and IIb/d fiber types of the mixed gastrocnemius muscles is shown in Figure 3. Significant group by time interactions existed in all fiber types (all P<0.05). The type I fiber cross-sectional area was less in Px vs. Shams at both 4 and 8 weeks (both P<0.05) but both groups had significant increases in fiber area from 4 to 8 weeks (both P<0.05). Similarly, type IIa fiber cross-sectional area was less in Px vs. Shams at both 4 and 8 weeks (both P<0.05) and these fibers also continued to grow with time in both groups (both, P<0.05). Finally, type IIb/d fiber areas were lower in Px than Shams at both 4 and 8 weeks (both P<0.05) and only grew significantly in the Shams (P<0.05). The findings of reduced cross sectional areas in all fiber types in the mixed gastrocnemius muscles and severely attenuated growth of the type IIb/d fibers in Px rats was consistent with measures made in the plantaris muscles (data not shown).

### Oxidative Enzyme Activity

Muscle oxidative markers (COX and SDH activity) were measured at the 8 week time point and were consistently lower in diabetic rat muscle. Specifically, COX activity in the GP was lower in Px (6.72±0.49 µmole/min/g) than in Shams (10.6±1.1 µmole/min/g) (P<0.05). SDH activity, as measured by histological staining in the mixed gastrocnemius, was also significantly lower in Px compared to Shams in all fiber types (Sham vs. Px, respectively-type I; 181±12 vs. 152±16; type IIa; 206±9.4 vs. 183±15; type IIb/d 174±11 vs. 152±13% Relative Optical Density). This finding was consistent with measures made in the plantaris muscle (data not shown).

### mTOR Signaling, Fractional Rates of Protien Synthesis and Protein Ubiquitination

**Figure 4:**
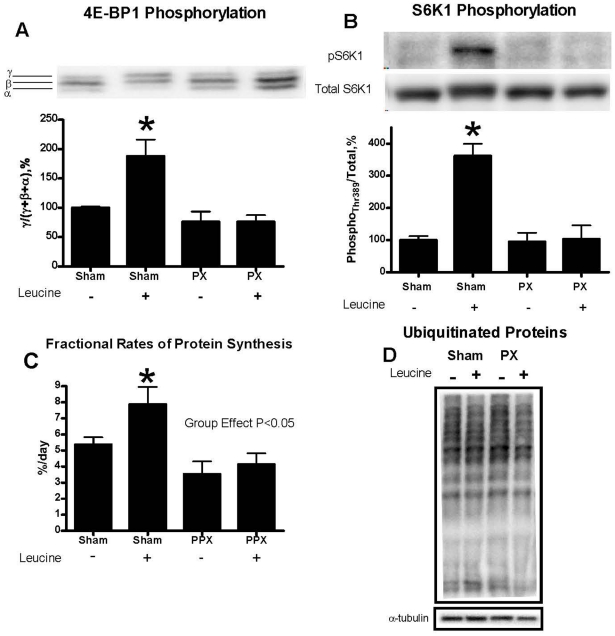
Phosphorylation of 4E-binding protein 1 (4E-BP1) (A) and S6K1 (threonine 389) (B) with, or without, leucine gavage, fractional protein synthesis rates (C) and total ubiquinated proteins (D) in gastrocnemius muscle of Sham and Px rats. Muscles were harvested 30 minutes following a gavage in the fasted state of either leucine (+) or water (−) at 4 weeks post surgery (for further details, see methods). On phosphorylation, 4E-BP1 migrates with different mobilities (i.e. α,β,γ, the γ-form being the most phosphorylated.) Data are expressed as the fraction of 4E-BP1 in the γ-form to the total of all 4E-BP1 forms, expressed as a percentage of Sham rats with water gavage. S6K1 (threonine 389) phosphorylation is also expressed as percentage of Shams with water gavage. Fractional protein synthesis rates, expressed as a percentage per 24 hours, was lower in Px than in Shams (main effect of group, P<0.05) and increased in response to leucine gavage in Shams but not in Px animals. Total ubiquitinated proteins, normalized to α-tubulin, in gastrocnemius muscle 30 minutes following either water or leucine gavage are expressed relative to Shams. Ubiquitinated protein levels did not change significantly with leucine gavage and so the data were pooled into Sham and Px groups for statistical analysis. * P<0.05 vs Sham (−).

As mentioned above, at 4 weeks post surgery, another group of Sham (n = 6) and Px (n = 8) rats were fasted for ∼18 hours and subjected to either oral gavage of L-leucine or distilled water. The animals were then killed 30 minutes later for the determination of key markers of protein synthesis and ubiquitination. 4EBP-1 and S6K1 phosphorylation in mixed gastrocnemius muscle increased 2-fold and 3.5-fold, respectively, following leucine gavage in Shams and was unchanged in Px rats (both P<0.05, Figures 4A and 4B).

In another subset of identically treated animals (n = 8 Shams; 6 Px), fractional protein synthesis rates, expressed as a percentage of amino acid incorporation into muscle protein per day, were measured 4 weeks post surgery by injection of [3H]phenylalanine tracer either with or without leucine gavage. Protein synthesis rates were significantly lower in Px than in Shams (main effect of group P<0.05) and only increased with leucine gavage in the Shams (Figure 4C, P<0.05). Finally, ubiquitinated protein levels, when measured in the fasted state, were similar between groups and were unchanged by leucine feeding (Figure 4D).

## Discussion

In this study, we show that muscle growth and contractile force production are profoundly impaired in the Px rodent model of adolescent T1DM and that this impairment is likely as a result of reduced rates of muscle protein synthesis. Specifically, we found that the skeletal muscle growth of all fiber types (type I, IIa, IIb/d), and overall force production of the gastrocnemious/plantaris complex, are dramatically attenuated at 4 and 8 weeks after the onset of hypoinsulinemia/hyperglycemia in growing Px rats compared with Sham-surgery rats. Furthermore, our results indicate that indices of protein synthesis (i.e. mTOR signaling and fractional rates of protein synthesis) are dramatically reduced in growing T1DM muscle with no observable differences in protein degradation, at least when measured in the fasted state (as assessed by global protein ubiquitination in fasted and leucine fed animals). The profound changes in young skeletal muscle exposed to the negative environment of uncontrolled T1DM suggests that early detection and intervention is of critical importance in pediatric populations to ensure that optimal muscle growth is not jeopardized, thereby affecting the potential for muscle mass accumulation and long-term physical capacities.

Long-standing, poorly controlled T1DM results in a number of disease-related complications including neuropathy, nephropathy, retinopathy and cardiovascular disease [1]. Another complication of T1DM is muscle weakness and reduced skeletal muscle mass, a complication that may be termed clinically as a “myopathy”, although some debate exists if this term is appropriate as skeletal muscle force production per unit of mass (or cross sectional area) may not necessarily be impaired in persons with T1DM [20]. While a myopathy may be particularly evident in patients with long-standing disease who have clinically detectible peripheral neuropathy [21]–[24], evidence also exists that children and young adults with short-term T1DM exhibit reduced physical capacities [25]–[30], impaired growth velocities [31], [32] and abnormalities in their GH–IGF-I axis [33], even when anthropometrically- and/or activity-matched. These findings suggest that a lower muscle volume may exist in young people with T1DM well before other micro- and macrovascular complications arise.

What remains to be fully elucidated are the effects of untreated or poorly controlled T1DM on adolescent skeletal muscle growth, function and phenotype, at a time when muscle growth rates normally would peak. The rate of whole body protein degradation and amino acid oxidation are enhanced in adolescents with T1DM compared to nondiabetic adolescents [34] and insulin therapy has been shown to reduce rates of protein degradation but not increase rates of protein synthesis in adolescents with the disease [35]. To date, however, few studies have investigated the effects of adolescent T1DM on skeletal muscle and contractile performance in T1DM, although some rodent data suggests that muscle protein synthesis rates may be impaired in insulin deficient animals (for a review see [36] and [20]). In a recent study, we compared the diabetic myopathy in the *Ins2^Akita^* ^+/−^ murine model of T1DM with the commonly used streptozotocin model and found several important differences in muscle characteristics [14]. However, we did not examine muscle growth in that study *per se*, as only one time point was assessed (8 weeks following diabetes induction) and no measures of protein synthesis were performed. While earlier studies attempted to define the mechanisms that contribute to impaired structure and function of skeletal muscle with T1DM, streptozotocin (STZ) administration was the diabetogenic agent was frequently used to induce hypoinsulinemia/hyperglycemia [37]–[45]. The use of the STZ model for the investigation of T1DM myopathy may lead to incorrect conclusions, however, as STZ, a potent DNA methylating agent, has been shown to have direct effects on skeletal muscle independent of hyperglycemia/hypoinsulinemia [46].

The effects of T1DM on overall growth in humans are well documented [47], as is the physiological effects of insulin on muscle growth [36]. There is a blunted pubertal growth spurt as evidenced by reduced peak height velocity standard deviation scores in insulin treated boys and girls with T1DM [48]–[51]. The effects on muscle growth, *per se*, in adolescents with the disease have yet to be fully clarified. In this study, we observed dramatic attenuation in overall growth, as measured by total body mass, muscle mass, fat mass and skeletal muscle length, between 4 week old and 13 week old Px rats, which corresponds to the adolescent period in rats. That these rodents are still in the growth phase of development is particularly evident in Sham operated rats given the large increases in body mass, muscle masses and fiber areas throughout the experimental protocol (Figures 1 and 3, Table 1).

Although growth was impaired in all fiber types of the Px animals in this study, we found that type II fibers, particularly the IIb/d form, were considerably more susceptible to attenuations in growth rate (Figure 3). In fact, we failed to measure any significant increase in type IIb/d fiber area between 4 and 8 weeks in the Px animals, while these same fibers nearly doubled in size in the Shams. These observations are consistent with reports of smaller type II fiber areas and muscle mass in other models of T1DM [14], [38], [52]. Together, these data suggest that the diabetic environment, at least under the conditions of this study (i.e. severe hyperglycemia/hypoinsulinemia and sedentary behavior) has greater deleterious effects on type II compared with type I fibers. By looking at this early time course of disease progression, we have extended these previous observations of reduced muscle mass size and have shown that the impairment in muscle growth in type II fibers with this disease is likely most dramatic during adolescence. Specifically, during pre adolescence (i.e. time 0 in this study, age 4 weeks in rats), all muscle fibers are of similar size and the relative reductions in fiber area growth between pre adolescents to adolescents (from 0 to 4 weeks) in the Px rats are consistent across fiber types (Figure 3). However, during later stages of adolescence, it has been reported that type I fibers do not increase dramatically in size, while type II fibers, particularly the type IIb/d fibers, undergo significant hypertrophy [53]. Our findings clearly indicate that if muscle is exposed to the T1DM environment during the critical stages of adolescence, a dramatic impairment in type II fiber growth will occur.

An alternative explanation exists for the more profound affect of T1DM on type IIb/d fiber growth in the conditions of this study (i.e. sedentary cage conditions). It is important to note that sedentary cage living in rodents likely recruits primarily the type I and IIa muscle fibers and not type IIb/d fibers. Moreover, it is well established that mechanical loading (i.e. exercise) overcomes the requirement of insulin to increase muscle mass in all muscle fiber types [54]. Thus, conceivably, it may be that the attenuation in growth of the type IIb/d fibers in the Px rats is largely because these rats were primarily sedentary in nature and not using these fast twitch muscle fibers on a regular basis. This finding may be clinically important as it suggests that newly diagnosed T1DM adolescents are at increased risk for impaired muscle growth and development, particularly if they are sedentary. Importantly, reduced type IIb fiber mass accumulation has been implicated in the deterioration of insulin sensitivity and body composition in rodents [55], both of which would place the diabetic individual at a further disadvantage from a glycemic management point of view. Whether or not the impairment in muscle growth can be normalized by intensive insulin therapy or by increased physical activity or a combination of these treatments requires urgent investigation.

The mechanisms for impaired muscle growth in T1DM are not entirely clear. The reduced insulin concentrations in T1DM would be expected to directly impair muscular protein synthesis in response to feeding and could be the major contributing factor to the phenotype observed in human and animal models of the disease [56]. As expected, we observed considerably lower fed plasma insulin levels in Px rats; levels that mimic that of prolonged fasting in Sham animals (Table 2) and muscle protein synthesis rates in the Px rats were about 50% of that observed in Shams after leucine feeding (Figure 4C). Protein feeding, and the resultant rise in circulating insulin levels in healthy individuals, is thought to be important in limiting protein breakdown and maintaining muscle mass during growth and development in humans with T1DM [57] and is directly involved in activating protein synthesis through the mTOR signaling pathway. Both 4E-BP1 and S6K1 are downstream phosphorylation targets of mTOR and stimulate mRNA translation initiation in the post-absorptive state. The initiation phase of mRNA translation plays a pivotal role in the regulation of protein synthesis [58]. We found that following leucine gavage, which simulates protein feeding in rats, there was virtually no increase in phosphorylation status of these key synthesis markers in the Px rats (Figures 4A and 4B). This finding is in line with the observation that insulin plays an important role in the activation of mTOR signaling following amino acid intake in muscle [59]. Taken together, our results provide strong evidence that the attenuation in muscle growth in Px rats was related to a down regulation of the mTOR signaling cascade and reduced rates of protein synthesis in response to nutrient feeding. However, it is important to note that some muscle protein synthesis did still occur during adolescence in this animal model of T1DM, as evidenced by the increase in muscle mass, fiber areas and fractional rates of protein synthesis, perhaps independent of insulin signaling and 4E-BP1 or S6K1 phosphorylation, as has been suggested previously [59]. Our observations that despite hyperphagia, circulating insulin levels remain in a fasted state in Px rats are also in support of the hypothesis that insulin deficiency in this model explains much of the attenuation in body mass gain and muscle mass growth. Nevertheless, other disturbances in metabolism, such as elevations in glucocorticoids (Table 2), increases in interleukin-6 levels or reductions in insulin-like growth factor-1 may also play a role in attenuating muscle growth in T1DM [20].

The reduced muscle mass, due to impaired growth of all fiber types, likely accounts for the lower absolute force production observed at 4 weeks of diabetes. Indeed, when expressed per unit of muscle mass, Fmax was similar between Px and Sham animals both at 4 weeks and 8 weeks. However, with continued exposure to the T1DM environment, the attenuated growth of type II fibers in the Px animals likely accounts for the inability of their muscles to generate any increase in absolute maximal force production between the 4 and 8 week time period. In comparison, the muscles from Sham rats displayed a 50% increase in maximal tetanic force production from 4 to 8 weeks, likely as a result of their dramatic increase in their type II fiber cross sectional area. A recent investigation into the muscle contractile characteristics of *Ins2^Akita^* ^+/−^ mice revealed similar reductions in maximal force capacity relative to control mice [14]. Consistent with this prior work, we found that relative peak force production was normal in T1DM rats as were the contractile dynamics when expressed relative to Fmax, indicating that attenuated muscle growth is the primary contributor to reduced force production, and not a specific effect of T1DM on the myofibrillar apparatus. Taken together, these observations help to explain the documented decrease in skeletal muscle performance in young people with T1DM when compared to their age-matched peers [60], [61].

Somewhat surprisingly, we observed reductions in muscle twitch time to peak tension and half-relaxation time at 8 weeks, but not at 4 weeks, in Px vs. Sham rats (Table 3). This apparent increase in twitch performance would normally indicate an increase in fast myosin properties (i.e. type IIb/d fibers), which is indeed counter to what we have speculated to have occurred in these animals. It may be that the small reduction in peak twitch amplitude at 8 weeks might explain the reduced contraction and relaxation time in these animals, although an increase in the number of type II fibers (although with reduced fiber areas) with T1DM, as has been observed in human studies of T1DM [62], [63], can not be ruled out.

Consistent with studies using STZ rodents that report reductions in oxidative capacity [62], [64], [65], we also found reductions in SDH and COX activity in the GP muscles in the 8 week Px group. Despite these reductions in oxidative enzymes, we observed attenuation in relative muscular fatigue rates at both 4 and 8 weeks after Px- compared to Sham- surgery (Figure 2 inset). However, when the 8 week fatigue protocol traces are viewed in absolute terms (Figure 2), a dramatic reduction in force generation is initially observed in the Sham-surgery rats compared with Px. We would speculate that fatigue of the larger mass of IIb/d (fast glycolytic) fibers in the Sham group, relative to Px, accounted for this precipitous drop and therefore, when made relative to initial force would be viewed as a fatigue-resistance in Px animals. While this hypothesis requires confirmation, support comes from our previous work in *Ins2^Akita+/−^* mice that showed no difference in relative fatigue rates between control and 8 week diabetic mice when a low-frequency fatigue protocol (2 min; 2 Hz) was utilized [14]. The low-frequency fatigue protocol in that previous study would have likely been of insufficient magnitude to elicit fatigue in type II fibers.

Our Px model of T1DM clearly has some limitations that should be discussed. While it may be speculated that removal of acinar cells belonging to the exocrine portion of the pancreas could account for reductions in body/tissue mass accumulation, it has been reported previously that following 90% pancreatectomy the digestive function of the pancreas is well maintained [66]–[68]. Moreover, the reduction in body mass observed in our Px animals is consistent with the ∼20% reduction in mass observed in hyperglycemic *Ins2^Akita^* ^+/−^ mice [14]. We also found that direct leucine gavage (which would not require digestive enzymes for intestinal absorption) resulted in an impaired response in mTOR signaling in Px rats, which suggests that a relative reduction in digestive enzymes had a minimal effect on the impaired muscle growth in these animals. Finally, as pointed out in the above discussion, the observations made in this study may only pertain to young Px rats who are not treated with exogenous insulin or allowed physical activity, unlike what is typically done in the clinical care of young patients with T1DM. As such, the relevance that this study has to humans with T1DM remains to be established.

In summary, we found that adolescent T1DM skeletal muscle is severely impaired in its capacity for growth, particularly if this occurs at a time when type II fiber development is taking place. The impaired growth can account for the impairments in force production, as force generation made relative to muscle mass was not different between groups. Unlike the other micro- and macrovascular complications associated with long standing diabetes, these differences in muscle growth and resultant decrements in contractile performance exist early on in the disease process. Our data point to impairments in protein synthesis, at a time when these pathways would normally be accelerated. Given that optimal growth is a major goal in the intensive treatment of T1DM children, these results should aid in defining new therapeutic strategies to ensure proper skeletal muscle growth and maximize skeletal muscle mass into adulthood.

## Supporting Information

Figure S1 Force frequency curves in Px and Sham groups at 4 and 8 weeks, expressed relative to Sham Fmax values (upper panels) and to the Fmax values in each group (lower panels).
(0.48 MB TIF)

## References

[1] The Diabetes Control and Complications Trial Research Group. (1993). The effect of intensive treatment of diabetes on the development and progression of long-term complications in insulin-dependent diabetes mellitus.. N Engl J Med.

[2] Donaghue KC, Chiarelli F, Trotta D, Allgrove J, Dahl-Jorgensen K (2009). Microvascular and macrovascular complications associated with diabetes in children and adolescents.. Pediatr Diabetes.

[3] Hamilton J, Daneman D (2002). Deteriorating diabetes control during adolescence: Physiological or psychosocial?. J Pediatr Endocrinol Metab.

[4] Mozdziak PE, Pulvermacher PM, Schultz E (2000). Unloading of juvenile muscle results in a reduced muscle size 9 wk after reloading.. J Appl Physiol.

[5] Darr KC, Schultz E (1989). Hindlimb suspension suppresses muscle growth and satellite cell proliferation.. J Appl Physiol.

[6] Reske-Nielsen E, Harmsen A, Vorre P (1977). Ultrastructure of muscle biopsies in recent, short-term and long-term juvenile diabetes.. Acta Neurol Scand.

[7] Jakobsen J, Reske-Nielsen E (1986). Diffuse muscle fiber atrophy in newly diagnosed diabetes.. Clin Neuropathol.

[8] Reske-Nielsen E, Gregersen G, Harmsen A, Lundbaek K (1970). Morphological abnormalities of the terminal neuromuscular apparatus in recent juvenile diabetes.. Diabetologia.

[9] Reske-Nielsen E, Lundbaek K, Gregersen G, Harmsen A (1970). Pathological changes in the central and peripheral nervous system of young long-term diabetics. the terminal neuro-muscular apparatus.. Diabetologia.

[10] Bonner-Weir S, Trent DF, Weir GC (1983). Partial pancreatectomy in the rat and subsequent defect in glucose-induced insulin release.. J Clin Invest.

[11] Burke RE, Levine DN, Tsairis P, Zajac FE (1973). Physiological types and histochemical profiles in motor units of the cat gastrocnemius.. J Physiol (Lond).

[12] Gordon JW, Rungi AA, Inagaki H, Hood DA (2001). Effects of contractile activity on mitochondrial transcription factor A expression in skeletal muscle.. J Appl Physiol.

[13] Ogilvie RW, Feeback DL (1990). A metachromatic dye-ATPase method for the simultaneous identification of skeletal muscle fiber types I, IIA, IIB and IIC.. Stain Technol.

[14] Krause MP, Riddell MC, Gordon CS, Imam SA, Cafarelli E (2009). Diabetic myopathy differs between Ins2Akita+/− and streptozotocin-induced type 1 diabetic models.. J Appl Physiol.

[15] Crozier SJ, Kimball SR, Emmert SW, Anthony JC, Jefferson LS (2005). Oral leucine administration stimulates protein synthesis in rat skeletal muscle.. J Nutr.

[16] Adegoke OA, Chevalier S, Morais JA, Gougeon R, Kimball SR (2009). Fed-state clamp stimulates cellular mechanisms of muscle protein anabolism and modulates glucose disposal in normal men.. Am J Physiol Endocrinol Metab.

[17] Garlick PJ, McNurlan MA, Preedy VR (1980). A rapid and convenient technique for measuring the rate of protein synthesis in tissues by injection of [3H]phenylalanine.. Biochem J.

[18] Suzuki O, Yagi K (1976). A fluorometric assay for beta-phenylethylamine in rat brain.. Anal Biochem.

[19] Attaix D, Ventadour S, Codran A, Bechet D, Taillandier D (2005). The ubiquitin-proteasome system and skeletal muscle wasting.. Essays Biochem.

[20] Krause MP, Riddell MC, Hawke TJ (2010). Effects of type 1 diabetes mellitus on skeletal muscle: Clinical observations and physiological mechanisms..

[21] Andersen H, Poulsen PL, Mogensen CE, Jakobsen J (1996). Isokinetic muscle strength in long-term IDDM patients in relation to diabetic complications.. Diabetes.

[22] Andersen H, Gadeberg PC, Brock B, Jakobsen J (1997). Muscular atrophy in diabetic neuropathy: A stereological magnetic resonance imaging study.. Diabetologia.

[23] Andersen H (1998). Muscular endurance in long-term IDDM patients.. Diabetes Care.

[24] Andersen H, Gjerstad MD, Jakobsen J (2004). Atrophy of foot muscles: A measure of diabetic neuropathy.. Diabetes Care.

[25] Poortmans JR, Saerens P, Edelman R, Vertongen F, Dorchy H (1986). Influence of the degree of metabolic control on physical fitness in type I diabetic adolescents.. Int J Sports Med.

[26] Huttunen NP, Kaar ML, Knip M, Mustonen A, Puukka R (1984). Physical fitness of children and adolescents with insulin-dependent diabetes mellitus.. Ann Clin Res.

[27] Baraldi E, Monciotti C, Filippone M, Santuz P, Magagnin G (1992). Gas exchange during exercise in diabetic children.. Pediatr Pulmonol.

[28] Gusso S, Hofman P, Lalande S, Cutfield W, Robinson E (2008). Impaired stroke volume and aerobic capacity in female adolescents with type 1 and type 2 diabetes mellitus.. Diabetologia.

[29] Larsson Y, Persson B, Sterky G, Thoren C (1964). Functional adaptation to rigorous training and exercise in diabetic and nondiabetic adolescents.. J Appl Physiol.

[30] Sterky G (1963). Physical work capacity in diabetic schoolchildren.. Acta Paediatr.

[31] Bognetti E, Riva MC, Bonfanti R, Meschi F, Viscardi M (1998). Growth changes in children and adolescents with short-term diabetes.. Diabetes Care.

[32] Gunczler P, Lanes R (1999). Poor metabolic control decreases the growth velocity of diabetic children.. Diabetes Care.

[33] Rosa JS, Galassetti PR (2009). Altered molecular adaptation to exercise in children with type 1 diabetes: Beyond hypoglycemia.. Pediatr Diabetes.

[34] Caprio S, Cline G, Boulware S, Permanente C, Shulman GI (1994). Effects of puberty and diabetes on metabolism of insulin-sensitive fuels.. Am J Physiol.

[35] Caso G, McNurlan MA (2010). Effect of insulin on whole body protein metabolism in children with type 1 diabetes.. Curr Opin Clin Nutr Metab Care.

[36] Miyazaki M, Esser KA (2009). Cellular mechanisms regulating protein synthesis and skeletal muscle hypertrophy in animals.. J Appl Physiol.

[37] Chonkar A, Hopkin R, Adeghate E, Singh J (2006). Contraction and cation contents of skeletal soleus and EDL muscles in age-matched control and diabetic rats.. Ann N Y Acad Sci.

[38] Cotter MA, Cameron NE, Robertson S, Ewing I (1993). Polyol pathway-related skeletal muscle contractile and morphological abnormalities in diabetic rats.. Exp Physiol.

[39] Fahim MA, el-Sabban F, Davidson N (1998). Muscle contractility decrement and correlated morphology during the pathogenesis of streptozotocin-diabetic mice.. Anat Rec.

[40] Lesniewski LA, Miller TA, Armstrong RB (2003). Mechanisms of force loss in diabetic mouse skeletal muscle.. Muscle Nerve.

[41] McGuire M, MacDermott M (1999). The influence of streptozotocin diabetes and metformin on erythrocyte volume and on the membrane potential and the contractile characteristics of the extensor digitorum longus and soleus muscles in rats.. Exp Physiol.

[42] Sanchez OA, Snow LM, Lowe DA, Serfass RC, Thompson LV (2005). Effects of endurance exercise-training on single-fiber contractile properties of insulin-treated streptozotocin-induced diabetic rats.. J Appl Physiol.

[43] Stephenson GM, O'Callaghan A, Stephenson DG (1994). Single-fiber study of contractile and biochemical properties of skeletal muscles in streptozotocin-induced diabetic rats.. Diabetes.

[44] Vignaud A, Ramond F, Hourde C, Keller A, Butler-Browne G (2007). Diabetes provides an unfavorable environment for muscle mass and function after muscle injury in mice.. Pathobiology.

[45] Goldberg AL (1979). Influence of insulin and contractile activity on muscle size and protein balance.. Diabetes.

[46] Johnston AP, Campbell JE, Found JG, Riddell MC, Hawke TJ (2007). Streptozotocin induces G2 arrest in skeletal muscle myoblasts and impairs muscle growth in vivo.. Am J Physiol, Cell Physiol.

[47] Chiarelli F, Giannini C, Mohn A (2004). Growth, growth factors and diabetes.. Eur J Endocrinol.

[48] Tattersall RB, Pyke DA (1973). Growth in diabetic children. studies in identical twins.. Lancet.

[49] Vanelli M, de Fanti A, Adinolfi B, Ghizzoni L (1992). Clinical data regarding the growth of diabetic children.. Horm Res.

[50] Du Caju MV, Rooman RP, op de Beeck L (1995). Longitudinal data on growth and final height in diabetic children.. Pediatr Res.

[51] Brown M, Ahmed ML, Clayton KL, Dunger DB (1994). Growth during childhood and final height in type 1 diabetes.. Diabet Med.

[52] Armstrong RB, Gollnick PD, Ianuzzo CD (1975). Histochemical properties of skeletal muscle fibers in streptozotocin-diabetic rats.. Cell Tissue Res.

[53] Oertel G (1988). Morphometric analysis of normal skeletal muscles in infancy, childhood and adolescence. an autopsy study.. J Neurol Sci.

[54] Spangenburg EE, Le Roith D, Ward CW, Bodine SC (2008). A functional insulin-like growth factor receptor is not necessary for load-induced skeletal muscle hypertrophy.. J Physiol.

[55] Izumiya Y, Hopkins T, Morris C, Sato K, Zeng L (2008). Fast/Glycolytic muscle fiber growth reduces fat mass and improves metabolic parameters in obese mice.. Cell Metab.

[56] Anthony JC, Lang CH, Crozier SJ, Anthony TG, MacLean DA (2002). Contribution of insulin to the translational control of protein synthesis in skeletal muscle by leucine.. Am J Physiol Endocrinol Metab.

[57] Charlton M, Nair KS (1998). Protein metabolism in insulin-dependent diabetes mellitus.. J Nutr.

[58] Shah OJ, Anthony JC, Kimball SR, Jefferson LS (2000). 4E-BP1 and S6K1: Translational integration sites for nutritional and hormonal information in muscle.. Am J Physiol Endocrinol Metab.

[59] Anthony JC, Reiter AK, Anthony TG, Crozier SJ, Lang CH (2002). Orally administered leucine enhances protein synthesis in skeletal muscle of diabetic rats in the absence of increases in 4E-BP1 or S6K1 phosphorylation.. Diabetes.

[60] Riddell MC, Iscoe KE (2006). Physical activity, sport, and pediatric diabetes.. Pediatr.Diabetes.

[61] Fricke O, Seewi O, Semler O, Tutlewski B, Stabrey A (2008). The influence of auxology and long-term glycemic control on muscle function in children and adolescents with type 1 diabetes mellitus.. J Musculoskelet Neuronal Interact.

[62] Fritzsche K, Bluher M, Schering S, Buchwalow IB, Kern M (2008). Metabolic profile and nitric oxide synthase expression of skeletal muscle fibers are altered in patients with type 1 diabetes.. Exp Clin Endocrinol Diabetes.

[63] Crowther GJ, Milstein JM, Jubrias SA, Kushmerick MJ, Gronka RK (2003). Altered energetic properties in skeletal muscle of men with well-controlled insulin-dependent (type 1) diabetes.. Am J Physiol Endocrinol Metab.

[64] Klueber KM, Feczko JD (1994). Ultrastructural, histochemical, and morphometric analysis of skeletal muscle in a murine model of type I diabetes.. Anat Rec.

[65] Snow LM, Lynner CB, Nielsen EM, Neu HS, Thompson LV (2006). Advanced glycation end product in diabetic rat skeletal muscle in vivo.. Pathobiology.

[66] Mizumoto R, Kawarada Y, Goshima H, Tamaki H, Sekoguchi T (1982). Carbohydrate metabolism and endocrine function in the pancreas remnant after major pancreatic resection.. Am J Surg.

[67] Sommer H (1987). Functional recovery of the exocrine pancreas in rats after partial resection.. Eur Surg Res.

[68] Yasugi H, Mizumoto R, Sakurai H, Honjo I (1976). Changes in carbohydrate metabolism and endocrine function of remnant pancreas after major pancreatic resection.. Am J Surg.

