# Supplementary figures and images for: Impaired Growth and Force Production in Skeletal Muscles of Young Partially Pancreatectomized Rats: A Model of Adolescent Type 1 Diabetic Myopathy?
Source: PLoS One. 2010 Nov 17;5(11):e14032. doi: 10.1371/journal.pone.0014032 (PMC2984438; doi:10.1371/journal.pone.0014032)

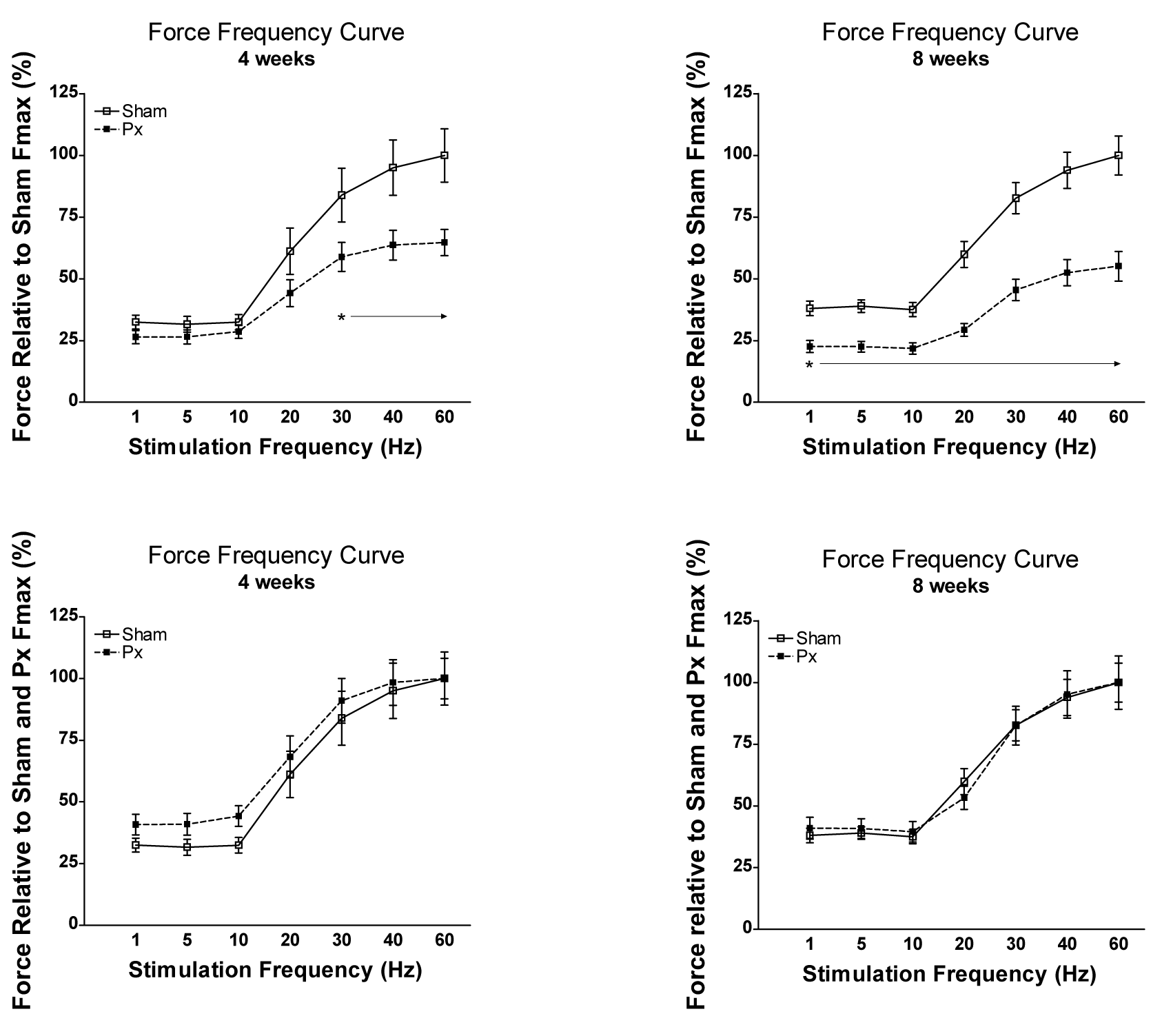

Supplement: Figure S1 — Force frequency curves in Px and Sham groups at 4 and 8 weeks, expressed relative to Sham Fmax values (upper panels) and to the Fmax values in each group (lower panels). (0.48 MB TIF) [file pone.0014032.s001.tif]
